# The molecular mechanisms of platelets in tumor invasion and metastasis: a controversial role

**DOI:** 10.1080/15384047.2026.2692154

**Published:** 2026-08-11

**Authors:** Amir Hossein Kheirkhah, Tahereh Zarei Taher, Zahra Khosrowpour, Omid Mahmoudian, Maria Kavianpour

**Affiliations:** a Student Research Committee, Qom University of Medical Sciences, Qom, Iran; b Department of Tissue Engineering and Applied Cell Sciences, Faculty of Medicine, Qom University of Medical Sciences, Qom, Iran; c Department of Pediatrics, University of Minnesota, Minneapolis, USA; d Faculty of Veterinary Medicine, Shahrekord University, Shahrekord, Iran; e Cellular and Molecular Research Center, Qom University of Medical Sciences, Qom, Iran

**Keywords:** Platelets, tumor metastasis, circulating tumor cells, cancer progression, targeted therapies, tumor growth

## Abstract

Platelets, traditionally recognized for their role in hemostasis, are now understood to actively contribute to tumor progression, angiogenesis, and metastasis. Through dynamic bidirectional interactions with cancer cells, platelets undergo measurable alterations in count, mean platelet volume, and molecular cargo, including proteins and mRNAs, supporting their potential utility as liquid biopsy biomarkers for early cancer detection. Within the tumor microenvironment, platelets contribute to immune evasion, support tumor cell intravasation and extravasation, enhance the survival of circulating tumor cells, and promote neovascularization. In addition, they can physically shield malignant cells from immune surveillance, thereby increasing metastatic potential. Although context-specific anti-tumor effects have been reported, the predominant evidence supports a largely pro-tumorigenic and pro-metastatic role for platelets. Elucidating the molecular mechanisms underlying platelet–tumor interactions is therefore essential for improving cancer risk stratification, prognostic evaluation, and therapeutic development. This review summarizes current advances in platelet-derived biomarkers and discusses emerging therapeutic strategies targeting platelet-mediated pathways to suppress metastasis and improve clinical outcomes in cancer patients.

## 1. Introduction

The sequence of events leading to the invasion and metastasis of tumor cells is of paramount importance in the prognosis of cancer patients.^1^ Tumor metastasis entails a sequence of events resulting in the development of secondary tumors in remote organs, significantly contributing to cancer-related mortality and morbidity.^2^ Metastatic cells subsequently penetrate the vascular basement membrane and extracellular matrix (ECM) via the circulatory system during extravasation. Ultimately, these cells adhere to a different site and multiply to form a secondary tumor.^3^ This series of events, known as the metastatic cascade, is essential for successful tumor dissemination. This metastatic cascade contributes to the complexity of cancer as a multifaceted disease.^4^ In the metastatic cascade, alterations in cell-cell and cell-matrix adhesion are critically significant. Platelets are crucial in hemostasis and thrombosis. They additionally facilitate tumor spread via interactions between platelets and tumor cells, which are linked to thrombus formation.^5^ Tumor cells stimulate platelets and alter platelet RNA patterns. Activated platelets cluster around circulating tumor cells (CTCs) to create a protective barrier that shields tumor cells from immune eradication.^6^

Platelets are small, specialized, non-nucleated blood cells. Numerous interacting actin filaments form the cytoskeleton that regulates platelet shape. The cytoplasm of these cells undergoes structural reorganization, resulting in the formation of granular platelet components, dense tubular networks, and a split membrane system.^7^ A glycoprotein membrane covers the surface of platelets, and their plasma membranes facilitate the exchange of small molecules and cytoplasmic contents with other cells, while also increasing the functional surface area for protein coagulation absorption.^6^ Alpha granules, dense granules, and a few lysosomes are all found in platelets. Alpha granules contain proteins such as pro- and anti-angiogenic factors, while dense granules carry calcium ions, 5-hydroxytryptamine (5-HT), adenosine triphosphate (ATP), and adenosine diphosphate.^8^ Platelet-derived exosomes and microparticles serve as biological messengers, delivering specific miRNAs, such as miR-21, miR-223, miR-339, and miR-328, with potential for tumor detection and diagnosis.^9^

In addition to their traditional function in coagulation, platelets exhibit a complex structural organization that enables their interactions with tumor cells. In addition to their traditional function in coagulation, platelets exhibit a complex structural organization that enables extensive interactions with tumor cells. A substantial body of evidence supports their involvement in multiple stages of cancer progression and metastasis including epithelial–mesenchymal transition (EMT), angiogenesis, anoikis resistance, extravasation, and metastatic niche formation.^5^ ^,^ ^10^ However, the strength and consistency of these mechanisms vary depending on tumor type, microenvironmental context, and immune status. While many platelet-mediated pathways are well established as pro-metastatic, emerging evidence also suggests that platelets may exert context-dependent or even tumor-suppressive effects under specific conditions, highlighting the complex and controversial nature of platelet biology in cancer. In this review, we summarize the molecular mechanisms underlying platelet–tumor interactions and critically discuss both established and emerging perspectives regarding the role of platelets in tumor invasion and metastasis.

## 2. Platelets in tumor invasion

### 2.1. Mechanisms of platelet-tumor cell interactions

Once in circulation, CTCs interact with platelets, activating them and causing tumor cell-induced platelet aggregation (TCIPA), a process that is widely recognized as a key mechanism supporting metastatic dissemination.^11^ TCIPA primarily consists of five key components: tissue factor (TF)-thrombin-protease-activated receptors (PARs),^12^ high-mobility group box 1 (HMGB-1)-Toll-like receptor 4 (TLR4),^10^ CD40L-CD40, TXA2-TXR, and ADP-P2Y12.^13^ Activated platelets secrete two of these, TXA2 and ADP, which further enhance TCIPA. Moreover, certain CTC subtypes release von Willebrand factor (vWF) or capture endothelial vWF. vWF attaches to the GPIbα subunit of the platelet glycoprotein Ib-IX-V (GPIb-IX-V), promoting platelet aggregation and adhesion.^11^

Vascular endothelial growth factors (VEGFs) are among the best-characterized mediators released following platelet–CTC interactions and are strongly associated with angiogenesis, vascular permeability, and metastatic niche formation.^14^ Upon activation by CTCs, platelets aggregate on their surface to form protective thrombi, aiding tumor cell survival. This process enables CTCs to circumvent shear-stress-induced obliteration by using platelets as a protective barrier.^15^ Conversely, this phenomenon reduces the surface exposure of CTCs, helping them avoid attack by natural killer (NK) cells.^16^ Platelets not only provide physical defense but also adhere their major histocompatibility complex class I (MHC-I) molecules to the CTC surface. When NK cells encounter these molecules on CTCs, they fail to recognize them, thereby enabling immune evasion. In addition, platelet-derived growth factors (PDGFs) directly inhibit NK cell cytotoxicity.^17^

**Figure 1.**
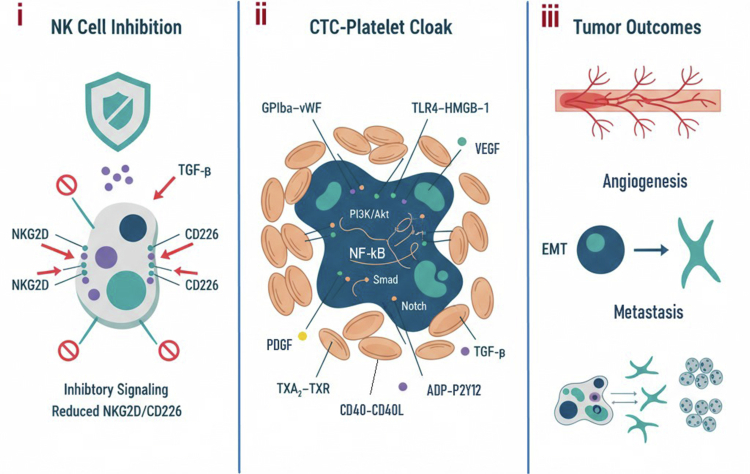
Schematic illustration of platelet–tumor cell crosstalk and downstream signaling in cancer metastasis. Circulating tumor cells (CTCs) activate platelets through multiple receptor–ligand axes, including GPIbα–vWF (von Willebrand factor), PARs (protease-activated receptors), thrombin, TLR4 (Toll-like receptor 4)–HMGB1 (high-mobility group box 1), CD40L–CD40, TXA₂ (thromboxane A₂)–TXR (thromboxane receptor), and ADP–P2Y₁₂. These interactions trigger tumor cell-induced platelet aggregation (TCIPA), leading to the formation of a platelet cloak around CTCs. This physical shield promotes immune evasion by: (i) transferring platelet-derived MHC-I molecules to the tumor cell surface, thereby masking tumor cells from NK cell recognition; (ii) releasing TGF-*β*, which suppresses NK cell cytotoxicity via downregulation of NKG2D and CD226 receptors; and (iii) facilitating CTC survival and protection from immune-mediated clearance within the circulation. Concurrently, platelet-derived mediators including VEGF, PDGF, and TGF-*β* activate intracellular signaling pathways in tumor cells (Smad, PI3K/Akt/mTOR, NF-κB, and Notch), driving epithelial–mesenchymal transition (EMT), angiogenesis, vascular permeability, and metastatic niche formation. Collectively, these mechanisms enhance CTC survival, extravasation, and colonization at distant sites.

By releasing soluble mediators, platelets impair NK cell–mediated antitumor activity. In particular, platelet-derived transforming growth factor-*β* (TGF-*β*) downregulates NKG2D and CD226 expression on NsK cells while reducing CD155 expression on CTCs, thereby weakening NK cell–tumor cell interactions.^14^ ^,^ ^18^ In parallel, platelet-derived factors such as TGF-*β* and PDGF contribute to EMT and metastatic progression through signaling pathways involving NF-κB, PI3K/Akt/mTOR, Notch-1, STAT-1, and p38/MAPK.^19-21^ These mechanisms are generally associated with pro-metastatic platelet activity; however, increasing evidence indicates that their biological impact may differ depending on tumor subtype, immune status, and microenvironmental context (Figure 1).

### 2.2. Extracellular vesicles: platelet-derived microvesicles and exosomes

Circulating platelets continuously secrete platelet-derived microvesicles (PMVs), which account for approximately 70% to 90% of hematogenous microvesicles in individuals with optimal health; however, the plasma concentration of PMVs exhibits considerable variability.^22^ Microvesicles (MVs) originate from membrane blebbing and carry cytoplasmic and membrane-associated proteins that reflect their cell of origin. Furthermore, platelets release exosomes, small vesicles derived from endocytic compartments that contain molecules produced by platelets.^23^ PMVs share both surface and internal features with activated platelets and act as mediators of platelet functions, while also possessing a unique ability to interact with different cell types. In contrast, PMVs do not contain *α*-granules or dense granules.^24^ Identifying the specific roles of PMVs in cancer progression is challenging due to their shared surface properties with platelets, technical challenges in isolating and distinguishing PMVs from other MVs, and the presence of common functional drivers, such as *P*-selectin, across various MV types, including those originating from cancer cells and the endothelium.^25^ PMVs may imitate activated platelets in promoting metastasis by displaying similar surface characteristics. Their membranes are enriched with activated platelet integrins and receptors, such as MHC-I and *P*-selectin, which facilitate the intravasation of tumor cells. Furthermore, many PMVs exhibit phosphatidylserine (PS) on their outer membranes, which enhances TF docking.^22^ ^,^ ^26^ Recent studies have demonstrated that PMVs transfer CXCR4, a chemokine receptor that activates signaling pathways that enhance the migration and survival of tumor cells in the bloodstream, to various solid cancer cell lines.^27^

PMVs can stimulate the expression of pro-metastatic proteins, such as metalloproteinases, enhancing invasiveness across various cancer types. This activity contributes to the increased metastatic foci observed in animal models, indicating that PMVs immediately facilitate the intravasation of metastatic neoplastic cells from the primary site and their subsequent dissemination to distant sites *in vivo.* ^28^ Furthermore, PMVs enhance the proliferation of metastatic tumor cells, facilitate adhesion to endothelial cells, and promote angiogenesis both *in vitro* and after PMV transfusion in rodents, reinforcing their role in functions previously attributed to platelets.^29^ An underexplored aspect of cancer metastasis is the function of PMVs in lymph, where they are highly concentrated, and their contribution to lymphangiogenesis, which is similar to vascular angiogenesis and is driven by growth factors contained within PMVs.^30^ ^,^ ^31^ PMVs in cancer exhibit complex immunomodulatory functions and may promote inflammatory signaling through interactions with immune cells such as neutrophils. These effects involve receptor transfer mechanisms and activation of pathways including NF-κB.^22^ ^,^ ^32^ PMVs can bridge leukocyte interactions, enhance inflammatory cytokine production, and exert proinflammatory effects in conditions such as arthritis. However, their specific role in cancer-related inflammation is unclear.^33^ ^,^ ^34^ PMVs also exhibit anti-inflammatory effects, modulating macrophages and dendritic cells toward less reactive states, promoting M2 macrophage differentiation, and reducing cytokine and chemokine secretion. Their roles in cancer immunity and implications for disease progression require further study.^35^ Accordingly, PMVs interact with cancer cells, endothelial cells, and immune cells, thereby significantly influencing tumor metastasis and immune responses, while ongoing research continues to elucidate their complex roles in cancer progression.

### 2.3. Platelet-induced modifications in the tumor microenvironment

Platelets are crucial in tumor progression, primarily through their interactions with the ECM.^36^ The ECM, a dynamic and complex network of proteins and molecules, offers structural and biochemical support to surrounding cells. During tumor progression, the ECM undergoes remodeling to promote tumor growth, invasion, and metastasis.^37^ The tumor microenvironment comprises not only cancer cells but also a diverse array of cell types, including epithelial and mesenchymal cells, vascular cells, and immune cells, all of which play pivotal roles in tumor proliferation.^38^ ^,^ ^39^ Recent evidence shows that platelets interact complexly with tumor microenvironment cells, often acting as supportive agents in tumor angiogenesis and metastasis. However, the extent and functional consequences of these interactions may differ across tumor types and microenvironmental settings, suggesting that platelet-mediated remodeling of the tumor microenvironment is highly context-dependent.^40^

Platelets are crucial to ECM remodeling by releasing various growth factors and cytokines that alter their composition and structure.^5^ Among these mediators, TGF-*β*, PDGF, and VEGF are considered key and relatively well-established contributors to platelet-driven tumor progression and metastatic remodeling. One key factor is TGF-*β*, which is known to trigger EMT in tumor cells, thereby enhancing their invasiveness and migration.^41^ Moreover, platelets promote the production of ECM components that are linked to poor prognosis, such as collagen and fibronectin, which are essential for maintaining tumor integrity and functionality.^42^ Additionally, the secretion of PDGF and VEGF by activated platelets plays a vital role in promoting angiogenesis and ECM production, thereby fostering a favorable environment for tumor growth.^5^ Platelets promote tumor cell invasiveness by facilitating ECM degradation. Pang’s group showed that blocking *P*-selectin and integrin αIIbβ3 inhibited platelet binding to tumor cells and prevented ECM degradation.^43^ Platelet-derived microvesicles (PMVs) have been demonstrated to encourage tumor cell invasiveness in certain cancer cell lines. Nevertheless, the magnitude and biological significance of PMV-mediated effects appear to vary among different tumor models and remain an active area of investigation. In a study by Janowska-Wieczorek et al., exposure of lung cancer cells to PMVs led to upregulation of matrix metalloproteinases (MMPs), key enzymes involved in extracellular matrix degradation and cancer metastasis. The magnitude of MMP induction varied among tumor cell lines, with more metastatic phenotypes exhibiting a stronger response to PMV stimulation.^44^ ^,^ ^45^ Finally, platelets play a vital part in inducing EMT in tumor cells. This process results in changes to the cytoskeleton, disruption of epithelial polarity, and decreased cell adhesion, which together enhance tumor cell motility and invasive potential. EMT increases *N*-cadherin levels while decreasing E-cadherin levels, facilitating cell detachment and promoting cancer growth and metastasis. Moreover, EMT contributes to the remodeling of the ECM, creating a more favorable microenvironment for the tumor.^46^

Angiogenesis is an essential mechanism in the malignancy and progression of tumors, playing an indispensable role in both solid and non-solid tumors. The involvement of platelets in angiogenesis has been recognized for a long time, from the initial phases through to the more advanced stages.^47^ ^,^ ^48^ Platelets release a variety of angiogenic regulators, some of which promote and others inhibit angiogenesis, highlighting the context-dependent nature of platelet involvement in tumor vascular remodeling. For instance, they store and release pro-angiogenic molecules like VEGF, basic fibroblast growth factor (bFGF), and PDGF. Conversely, they also possess anti-angiogenic regulators such as endostatin and Platelet Factor 4 (PF-4).^49^ Research shows that in addition to transporting and distributing angiogenic regulators produced by megakaryocytes, platelets selectively absorb angiogenic molecules from their surroundings, such as VEGF and endostatin.^50^ Studies have found associations between platelet counts and serum VEGF levels in patients with breast, colorectal, renal, and ovarian cancers, supporting the widely accepted role of platelets as major contributors to circulating angiogenic signaling in cancer.^51^ Platelet depletion in models of metastatic melanoma led to a significant reduction in tumor vessel density. Furthermore, treating human umbilical vein endothelial cells (HUVECs) with platelet-derived factors released from activated platelets enhanced endothelial cell tube formation. These findings further confirm the critical role of platelets in promoting angiogenesis and suggest their capacity to amplify tumor cells' angiogenic potential.^5^ ^,^ ^52^ ^,^ ^53^ Platelets regulate angiogenesis by releasing pro-angiogenic molecules, such as VEGF, or anti-angiogenic molecules, such as endostatin, depending on stimulation of protease-activated receptor-1 or 4 (PAR1 or PAR4).^54^ These findings suggest that platelet-mediated angiogenic responses are not uniform and may vary according to receptor-specific signaling and tumor microenvironmental conditions. Incubating platelets with thrombin, which activates PAR1 and PAR4, results in minimal release of VEGF or endostatin because the opposing pathways counteract each other. However, the releases from PAR1- and PAR4-stimulated platelets promote angiogenesis, with PAR1 exhibiting a stronger effect. While PAR4 stimulation may inhibit angiogenesis, it does not completely counteract platelets' overall positive impact. Additionally, co-incubating HUVECs with breast cancer cells enhances the formation of endothelial tubes, and the releases induced by PAR1 and PAR4 increase the ability of cancer cells to grow, with PAR1 having a more significant influence.^53^ The exact mechanism by which platelets regulate the release of various molecules remains poorly understood. Therefore, although many platelet-driven angiogenic pathways are well established, the regulatory mechanisms governing selective mediator release and their net effects on tumor progression remain areas of ongoing investigation.

There is speculation that regulators that are both pro- and anti-angiogenic are kept in separate *α*-granules, which are released in response to specific signals.^55^ A recent study using super-resolution immunofluorescence suggested that *α*-granules may contain opposing molecules distributed unevenly. Future research focused on the storage and release processes of platelet signaling molecules, as well as their interactions within different pathways, could improve our understanding of how platelets regulate angiogenesis.^56^ Altogether, these findings highlight the multifaceted roles of platelets in assisting tumor cell invasion, immune evasion, and microenvironmental remodeling, thereby positioning them as crucial but controversial players in the metastatic cascade.

## 3. Platelets in tumor metastasis

### 3.1. Platelets in initial migration and circulation

**Figure 2.**
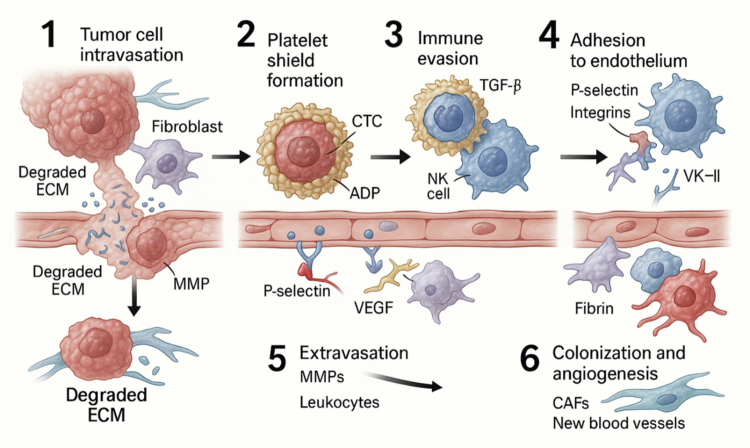
Platelet-mediated facilitation of the metastatic cascade: Illustration of the sequential stages in platelet-assisted tumor metastasis: (1) intravasation of tumor cells into the bloodstream, (2) platelet cloak formation and immune evasion, (3) endothelial adhesion and vascular interaction, (4) extravasation into distant tissues, and (5) colonization and metastatic niche formation supported by angiogenic and growth-promoting signals.

Tumor cells face multiple challenges upon entering the bloodstream, including mechanical stress, immune surveillance, and the harsh circulatory environment.^57^ However, they employ sophisticated mechanisms to overcome these barriers and ensure survival, often facilitated by interactions with platelets, immune cells, and endothelial cells. After entering the bloodstream, CTCs face high shear stress and immune defenses that threaten their integrity and survival.^58^ One key survival strategy of CTCs involves forming microaggregates with platelets, which provide a protective shield. This "platelet shield" is rapidly established when tumor cells activate platelets via factors such as TF and ADP, triggering coagulation cascades, as shown in Figure 2. ^59^ As detailed in Section 2.1, platelets facilitate immune evasion through physical shielding and molecular mimicry, including MHC-I transfer and secretion of immunosuppressive mediators such as TGF-*β*. This platelet “cloak” protects CTCs from NK cell-mediated lysis and mechanical stress during circulation.^60^ ^,^ ^61^ These mechanisms are widely regarded as central and well-established contributors to platelet-mediated metastatic dissemination. Moreover, platelet-driven fibrin clots enhance CTC survival and metastatic potential by protecting tumor emboli from NK cell attacks.^62^ Studies have demonstrated that disrupting platelet activation pathways or coagulation factors, such as prothrombin or fibrinogen, significantly reduces tumor survival at metastatic sites. Platelets also promote tumor cell adhesion to the vascular endothelium, a critical step in extravasation.^62^ Beyond their angiogenic effects, platelet-derived mediators also contribute to tumor cell extravasation during metastatic dissemination.^61^ ^,^ ^63^

Platelet-leukocyte interactions serve a crucial function in metastasis by linking thrombosis and immune responses.^64^ However, the immunological consequences of these interactions are highly context-dependent and may vary according to tumor type, inflammatory status, and immune microenvironment. These interactions occur through surface molecules and signaling factors, influencing tumor progression via several mechanisms: Platelets enhance neutrophil recruitment and activation, thereby regulating tumor inflammation and metastasis. Platelets interact with monocytes, enhancing their cytokine secretion and facilitating tumor-associated inflammation.^64^ Platelet-monocyte aggregates also support immune cell trafficking and extravasation. This interaction is critical for tumor progression, as it strengthens platelet-endothelium adhesion and stabilizes thrombi, ultimately supporting tumor growth and metastasis.^65^ Platelets influence macrophage polarization, driving monocytes toward either a tumor-promoting M2 phenotype or an anti-tumor M1 phenotype depending on the tumor microenvironment.^66^ Similarly, platelet-mediated regulation of dendritic cells and T-cell responses may exert either immunosuppressive or immune-supportive effects under different pathological conditions. Platelets modulate DC function, either enhancing or suppressing tumor antigen presentation and T-cell activation.^67^ T-Cell Modulation: Platelet interactions with CD4+ and CD8+ T-cells affect tumor progression, enhancing cytotoxicity or inducing regulatory T-cells that dampen immune responses.^67^ Platelets are essential for modulating humoral immunity by facilitating B-cell proliferation, antibody production, and isotype switching through CD40L-mediated signaling, thereby aiding the fight against tumors.^68^ NK Cell Suppression: Platelets suppress NK cell activity through mechanisms such as TGF-*β* secretion, reducing tumor cell recognition and cytotoxicity.^68^ These platelet-mediated interactions strengthen tumor-endothelial adhesion and promote thrombus formation, further facilitating metastatic progression.^65^ ^,^ ^66^ Nevertheless, emerging evidence suggests that certain platelet–immune interactions may also contribute to anti-tumor immune regulation in specific biological contexts, further emphasizing the controversial role of platelets in cancer progression.

### 3.2. Facilitating colonization of metastatic sites

Platelets are critical for supporting tumor cells in metastatic niches by enhancing immune evasion, tumor cell adhesion, and the creation of a favorable microenvironment.^69^ Many of these platelet-mediated functions are considered well-established drivers of metastatic progression, particularly in advanced solid tumors. Beyond the previously described platelet cloak mechanisms, TCIPA further enhances metastatic survival through additional NK cell–modulatory pathways, including Glucocorticoid-Induced TNF-Related Ligand (GITRL) signaling.^69^ Platelets play an active role in tumor extravasation by releasing cytokines, growth factors, and MMPs, which disrupt endothelial cell junctions, degrade extracellular matrix components, and increase vascular permeability.^70^ However, the relative contribution of these mechanisms may differ depending on tumor subtype and metastatic microenvironment. They also recruit granulocytes via cytokines such as CXCL5 and CXCL7, which enhance tumor cell retention in metastatic tissues. In metastatic niches, platelet interactions with cancer-associated fibroblasts (CAFs) and ECM components, such as fibronectin, promote tumor growth by secreting PDGF.^71^ At the same time, the balance between pro-angiogenic and anti-angiogenic platelet-derived mediators remains incompletely understood and continues to be actively investigated. Platelet-induced angiogenesis and lymphangiogenesis, mediated by factors like VEGF and VEGF-C, ensure tumor survival and nutrient supply.^71^ Creating new lymphatic vessels aids the spread of tumor cells to distant organs.^72^

Platelets release bioactive molecules such as TGF-*β*, PDGF, and VEGF, which initiate signaling pathways that enhance tumor viability, migration, and dissemination. TGF-*β* is particularly significant for inducing EMT, enhancing tumor cell invasion.^6^ Platelets interact directly with tumor cells, stimulating EMT and invasion. The activation of G-protein-coupled receptors (GPCRs) on platelets, such as P2Y1, P2Y12, and PAR receptors, triggers pathways such as NF-κB, which synergizes with TGF-*β* signaling to enhance metastasis.^73^ ^,^ ^74^ Platelet activation involves GPCRs and kinases, such as PI3K and PKC, which further promote metastasis by promoting platelet aggregation and the secretion of tumor-supportive molecules, such as ADP and thromboxane A2.^75^ Platelet-derived TGF-*β* suppresses NK cell activity, facilitating immune evasion and increasing metastatic potential.^76^ Furthermore, platelet-derived microparticles (PMPs) contribute to neovascularization by releasing TF and pro-coagulant molecules, supporting tumor growth and metastasis.^77^ Together, these multifaceted platelet functions critically support tumor cell survival, immune evasion, and metastatic colonization, highlighting their central, context-dependent, and controversial role in cancer progression.

## 4. The controversial role of platelets in cancer

Platelets are critical and increasingly recognized mediators in cancer biology, with a well-established role in promoting metastasis, commonly described as the “platelet cloak” phenomenon.^18^ However, emerging evidence indicates that their function is not strictly pro-tumorigenic, but rather highly context-dependent, influenced by tumor stage, tumor type, metastatic site, platelet activation status, and the surrounding tumor microenvironment. Therefore, platelet biology in cancer should be interpreted through an integrated, conditional framework rather than a binary classification.^6^ ^,^ ^78^ Therefore, platelet biology in cancer should be interpreted through an integrated, conditional framework rather than a binary classification.

### 4.1. The dominant pro-tumorigenic role

The prevailing evidence supports a strong pro-metastatic function of platelets in cancer progression. Upon activation by tumor cells via TCIPA, platelets release pro-tumorigenic mediators such as VEGF, bFGF, SDF-1, and MMPs, which promote angiogenesis and tumor dissemination.^79^ ^,^ ^80^ Platelets also play a key role in immune evasion. They protect CTCs from NK cells and cytotoxic T lymphocytes (CTLs) through multiple mechanisms: shielding tumor antigens, downregulating NK cell-activating receptors, such as NKG2D, presenting MHC-I molecules to engage NK cell inhibitory receptors, and producing immunosuppressive cytokines, such as TGF-*β*. Additionally, platelets express tumor necrosis factor (TNF) family ligands, including GITRL and Receptor Activator of Nuclear Factor Kappa-B Ligand (RANKL), which further dampen anti-tumor immunity.^61^ ^,^ ^81^ During metastasis, platelet adhesion receptors, including *P*-selectin, integrins, and GPVI, facilitate stable tumor–endothelial interactions that support secondary site colonization. Platelets secrete ATP and chemokines such as CXCL5 and CXCL7, creating a permissive microenvironment for extravasation and metastatic niche formation.^75^ ^,^ ^82^ Furthermore, platelets enhance resistance to anoikis, a type of programmed cell death induced by the loss of anchoring, which is activated through pro-survival pathways, including the TGF-β/Smad, Notch, and YAP1 signaling cascades.^83^

### 4.2. Evidence for anti-tumorigenic roles

Despite the well-established pro-metastatic functions of platelets, increasing evidence indicates that they may also exert anti-tumorigenic effects under specific biological contexts. In contrast, platelets also represent a major source of Angiopoietin-1 (Ang-1), which binds Tie2 receptors on endothelial cells and contributes to vascular stabilization. This process, often described as vascular normalization, may reduce endothelial permeability and potentially limit tumor cell extravasation during early stages of tumor progression.^13^ ^,^ ^84^ ^,^ ^85^ Under specific inflammatory conditions, platelets display TNF-related apoptosis-inducing ligand (TRAIL) on their surface. The interaction of TRAIL bound to platelets with death receptors (DR4/DR5) on susceptible tumor cells can trigger apoptosis, effectively eliminating circulating tumor cells before they can colonize distant organs.^86^ Experimental studies have shown that platelet transfusions can inhibit tumor cell proliferation by inducing G0/G1 cell cycle arrest. Platelet factor 4 (PF4), acting through the CXCR3B receptor, has been implicated in apoptosis induction and angiogenesis inhibition.^87^ ^,^ ^88^ Moreover, platelets may release microparticles that activate immune responses and hinder primary tumor growth. Thrombin-activated platelets (TAPLTs) have demonstrated the capacity to prevent tumor-induced angiogenesis in endothelial cells by targeting VCAM-1 and reducing reactive oxygen species (ROS).^89^ Interestingly, the tumor-modulating effects of platelets are influenced by several factors, including tumor type, anatomical location, microenvironmental conditions, and blood flow characteristics. In some experimental models, platelet depletion enhanced metastasis in certain organs while reducing it in others, suggesting that platelets may either advance or inhibit tumor dissemination, depending on the context.^90^ These findings collectively underscore the complexity of platelet involvement in cancer. While they are essential facilitators of metastasis and immune evasion, platelets can also exhibit inhibitory effects on tumor growth and vascularization. The dualistic nature of platelet function presents both a challenge and an opportunity for therapeutic intervention.^61^ Anti-platelet therapies, such as aspirin and tyrosine kinase inhibitors (TKIs), can reduce tumor progression but must be used with caution, given platelets' context-specific and sometimes protective roles.^82^ ^,^ ^91^ The diverse roles of platelets across distinct phases of the metastatic cascade, along with their key molecular mediators, are summarized in Table 1.

**Table 1.** Intentionally focuses on metastatic cascade (pro-tumor mechanisms).

Metastatic Stage	Platelet Function	Key Molecules/Mechanisms	Ref.
Tumor Cell Intravasation	Release of MMPs and growth factors	MMPs, VEGF, bFGF, SDF-1	[92]
Circulating Tumor Cell Protection	Shielding from immune cells, MHC-I presentation	TGF-*β*, PD-L1, *P*-selectin, cytokines	[93]
Adhesion to Endothelium	Mediating tumor cell-endothelial interaction	*P*-selectin, integrins, GPVI	[94]
Extravasation and Colonization	Promoting vascular permeability, survival	ATP secretion, CXCL5/CXCL7, TGF-β/Smad	[63]
Anoikis Resistance	Activation of pro-survival signaling pathways	Notch, YAP1, PI3K/AKT	[95,96]

### 4.3. Integrated context-dependent model of platelet function in cancer

The functional role of platelets in cancer is highly context-dependent and varies according to multiple interrelated biological determinants. Tumor stage represents a key factor, as platelet activity tends to be predominantly pro-tumorigenic in advanced and metastatic disease, where it facilitates dissemination and tumor progression, whereas in early-stage or micrometastatic settings platelets may contribute to vascular stabilization and immune containment.^69^ Tumor type also influences platelet behavior, with strong pro-metastatic evidence particularly in colorectal, gastric, and pancreatic cancers, while in other tumor entities the effects remain more variable and context-specific. In addition, the anatomical site of metastasis plays an important role; platelets promote pre-metastatic niche formation in organs such as the liver, lung, and bone, whereas in immune-active or well-perfused tissues their capacity to support dissemination may be restricted.^82^ The platelet activation state further modulates these effects, as hyperactivated platelets predominantly release pro-tumorigenic mediators such as VEGF, TGF-*β*, and MMPs, while physiological or transient activation can in some contexts trigger anti-tumor mechanisms including TRAIL-mediated apoptosis.^97^ Finally, the tumor microenvironment plays a major role in determining how platelets behave in cancer. In hypoxic, inflammatory, and immunosuppressive environments, platelets are more likely to promote metastasis and tumor progression. In contrast, in immune-active and more vascularly stable conditions, platelets may exhibit neutral or even protective effects. Together, these factors highlight that platelet function in cancer is dynamic and context-dependent, rather than strictly pro-tumor or anti-tumor.

## 5. Molecular mechanisms involved

### 5.1. Key platelet-associated signaling pathways

TGF-*β* is a crucial cytokine that plays a complex role in metastasis. *In vitro* studies using co-culture systems have shown that platelet-derived TGF-*β* downregulates NKG2D on NK cells, directly impairing their cytotoxic function.^98^ Notably, TGF-*β* demonstrates a bifunctional role in cancer progression: it functions as a nascent tumor suppressor, but paradoxically promotes tumor progression in advanced cancer stages.^98^ The activation of latent TGF-*β* is a multifaceted process involving enzymatic and non-enzymatic mechanisms, which can vary between tumor types and microenvironments.^99^ Sources of TGF-*β* within tumors include both cancer cells and stromal cells, contributing to context-dependent functional outcomes.^99^ *In vivo* animal models have validated causality; for instance, Labelle et al. showed in mice that platelet-derived TGF-*β* is essential for the EMT of circulating tumor cells and their effective extravasation. Additionally, research using transgenic mouse models indicates that epithelial TGF-*β* signaling reduces mammary tumor development, while loss of this pathway can increase pulmonary metastasis.^100^ ^,^ ^101^ The apoptotic response to TGF-*β* is modulated by proliferative states and microenvironmental signals, many of which remain to be elucidated.^100^ ^,^ ^102^ ^,^ ^103^ TGF-*β* signals through membrane-bound serine/threonine kinase receptors classified as Type II (TβRII) and Type I (TβRI) receptors.^104^ Ligand binding to TβRII initiates phosphorylation and activation of TβRI, which, in epithelial cells, predominantly involves Activin-Like Kinase 5 (ALK5).^104^ ^,^ ^105^ Activated TβRI mediates downstream signaling via Smad-dependent and Smad-independent pathways. The Smad pathway involves phosphorylation of receptor-regulated Smads (R-Smads), their nuclear translocation, and transcriptional regulation of target genes that modulate cell proliferation, differentiation, and apoptosis.^106^ ^,^ ^107^

In contrast, non-Smad pathways such as JNK/p38, Rho-like GTPases, MAPK/ERK, and PI3K/AKT contribute to tumor progression, especially in advanced stages. The JNK/p38 pathway, induced by TGF-*β* in response to cellular stress, is implicated in EMT and apoptosis.^108^ PI3K/AKT signaling enhances cellular viability and confers resistance to apoptosis, thereby supporting tumor growth and metastatic potential.^109^

**Figure 3.**
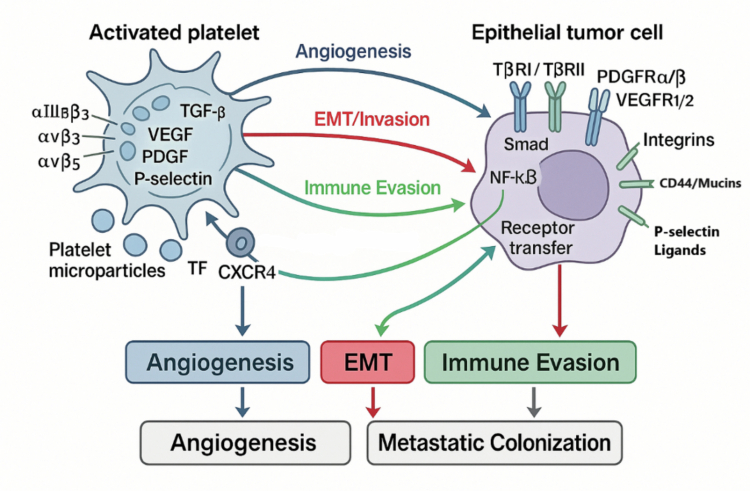
Key molecular pathways in platelet–tumor cell interactions. Platelets promote tumor progression through release of TGF-*β*, VEGF, and PDGF, activating EMT, angiogenesis, and metastatic niche formation. TGF-β/Smad signaling crosstalks with NF-κB and PI3K/Akt pathways to enhance invasion and immune evasion, while VEGF and PDGF support vascular and lymphatic remodeling. Platelet adhesion receptors (αIIbβ3, α6β1) and *P*-selectin mediate binding to tumor cells (e.g., CD44, mucins) and endothelium, facilitating extravasation and dissemination, with additional modulation by cancer-associated fibroblasts and immune cells.

The VEGF and PDGF pathways are central to tumor angiogenesis. VEGF family members, including VEGFA, VEGFB, VEGFC, VEGFD, and placental growth factor (PlGF), bind to tyrosine kinase receptors on endothelial cells to initiate angiogenic cascades.^110-112^ Isoforms VEGF121 and VEGF165 of VEGFA are particularly crucial in neovascularization within tumors and also regulate tumor cell survival and invasion through autocrine and paracrine signaling.^112^ *In vivo* studies using platelet-depleted mouse models have demonstrated a significant decrease in tumor vessel density, establishing a causal relationship between platelet function and angiogenesis.^113^ Human clinical data further support this notion; anti-VEGF therapies, such as Bevacizumab, are standard of care. Their efficacy is often influenced by platelet counts, emphasizing the clinical importance of this pathway.^8^ PDGF, secreted by platelets and stromal cells, serves as a significant mitogen for mesenchymal cells, including fibroblasts and smooth muscle cells. PDGF isoforms regulate cellular proliferation, differentiation, angiogenesis, and metastasis, largely via PDGFR-*α*-mediated signaling.^114^ Both VEGF and PDGF activate MAPK/ERK and PI3K/AKT pathways, which are attractive targets for anticancer therapies.^114^ Indeed, combination therapies involving VEGF inhibitors like bevacizumab have demonstrated efficacy in colorectal cancer by effectively suppressing tumor angiogenesis.^115^ The interconnections between TGF‑β/Smad, NF‑κB, PI3K/Akt, and pro‑angiogenic VEGF/PDGF signaling pathways, along with adhesion mechanisms, are illustrated in Figure 3.

### 5.2. Molecular interactions between platelets and tumor cells

Integrins are transmembrane adhesion receptors that facilitate connections between cells and the extracellular matrix, playing crucial roles in the development and metastasis of neoplasms.^116^ Dysregulated integrin signaling facilitates tumor initiation, growth, and dissemination. Specific integrin subtypes such as αvβ6 are expressed on activated endothelial cells and promote angiogenesis in pathological tissues. Integrins αvβ3 and αvβ5 have been shown to mediate angiogenesis induced by growth factors, including bFGF, TNF-*α*, TGF-*α*, and VEGF, and are key targets in the development of anti-angiogenic cancer therapies.^117^ *In vitro* flow chamber assays have characterized the binding kinetics of platelet integrins (αIIbβ3) to tumor cell surface receptors, such as CD44 and MUC1. These studies established a mechanism mediated by fibrinogen that acts as a “bridge.” *In vivo*, antibodies that block αIIbβ3 or *P*-selectin in mice have consistently inhibited metastatic seeding to the lungs, providing strong causal evidence. In addition, integrin signaling enhances angiogenesis and metastasis in hepatocellular carcinoma and confers breast cancer cells with resistance to chemotherapy-induced apoptosis.^75^ ^,^ ^116^ ^,^ ^117^ Integrins contribute to tumor progression by regulating endothelial cell activation, migration, survival, evasion of growth suppression, inflammation, metabolic reprogramming, immune evasion, and invasion through interactions with cancer-associated fibroblasts.^118^ ^,^ ^119^ Additionally, integrin-mediated drug resistance poses a major challenge in cancer treatment. Despite this, clinical trials targeting integrins α5β1, αvβ3, and αvβ5 have not yet yielded approved therapeutics, underscoring the complexity of integrin biology in cancer.^118^ ^,^ ^119^

*P*-selectin, a transmembrane protein stored in platelet alpha granules, translocates to the platelet surface upon activation, where it functions as a key adhesion receptor.^120^ It binds to ligands such as *P*-selectin glycoprotein ligand-1 (PSGL-1) on leukocytes and cancer cells, as well as to GPIb on platelets, facilitating platelet-tumor cell and platelet-endothelium interactions. *P*-selectin-mediated platelet aggregation accelerates tumor progression, as demonstrated in models of insulinoma, where blocking *P*-selectin interactions reduced tumor growth.^78^ ^,^ ^120^ Consequently, targeting *P*-selectin-dependent platelet-cancer cell adhesion is a promising therapeutic avenue under active investigation.^78^ ^,^ ^120^ These molecular pathways highlight the multifaceted role of platelets in supporting tumor progression, suggesting new avenues for therapeutic intervention targeting platelet-tumor interactions.^78^

### 5.3. Critical synthesis of mechanistic evidence

Synthesizing the wide range of studies presented, a clear hierarchical structure of evidence emerges regarding the interactions between platelets and tumors. At the foundational level, the primary role of platelets in shielding CTCs from high shear stress and NK cell cytotoxicity is the most robustly validated mechanism. This protective function is consistently supported by *in vitro* flow cytometry assays, *in vivo* murine models of metastasis, and clinical observations showing that thrombocytosis correlates with poor survival outcomes. Similarly, the adhesion cascade mediated by *P*-selectin and integrin αIIbβ3 is recognized as a consensus mechanism. Blocking these receptors leads to reproducible reductions in metastatic seeding in animal models. However, translating these findings into effective clinical therapies remains challenging due to associated hemostatic risks.^82^ ^,^ ^121^ The signaling roles of platelet-derived cytokines, particularly TGF-*β* and VEGF, are critically context-dependent. *In vitro* studies unequivocally demonstrate that these factors promote EMT and angiogenesis. However, the *in vivo* outcomes reveal a far more complex picture. TGF-*β* exhibits a biphasic nature: it functions as a tumor suppressor in the early stages but shifts to promote invasion in advanced stages. Consequently, platelets exert opposing effects depending on the disease phase. Furthermore, platelets serve as a significant source of pro-angiogenic VEGF, while also sequestering anti-angiogenic factors, such as endostatin, in distinct alpha granule populations. This indicates that the net effect on tumor vasculature is decisively shaped by the specific stimuli that trigger degranulation within the tumor microenvironment.^101^ ^,^ ^122^ Finally, emerging evidence points to a novel, hypothesis-generating layer of interaction: the horizontal transfer of functional biomolecules. Recent RNA-sequencing studies indicate that platelets can transfer specific miRNAs and spliced RNAs to tumor cells via microparticles, potentially reprogramming tumor metabolic or invasive profiles. While these mechanisms are fascinating, they are primarily grounded in high-resolution ex vivo and *in vitro* analyzes. Validation of their functional significance in clinical settings represents the next frontier of platelet oncology research.^123^ Ultimately, distinguishing between robustly validated mechanisms and those requiring further verification is crucial for clinical translation. A critical summary of these pathways, highlighting their respective levels of evidence and knowledge gaps, is provided in Table 2.

**Table 2.** Overview of platelet-mediated mechanisms in cancer progression.

Mechanism/Pathway	Primary Outcome	Level of Evidence	Consensus Status	Key Limitation/Gap	Ref.
TCIPA (*P*-selectin/Integrins)	Physical shielding of CTCs; Facilitated extravasation	High (In vitro, In vivo, Clinical correlations)	Well-Established	Clinical targeting (e.g., anti-*P*-selectin) is challenged by bleeding risks.	[101,124]
TGF-*β* Signaling	NK cell suppression; EMT induction	High (In vitro, In vivo)	Well-Established	Systemic TGF-*β* inhibition has pleiotropic side effects.	[125,126]
COX-1/Thromboxane A2	Platelet activation; Aggregation	High (Large Clinical Trials - e.g., Aspirin)	Well-Established	Benefit varies by tumor type (e.g., strong in GI cancers, weaker in others).	[127,128]
Angiopoietin-1/Vessel Normalization	Stabilization of tumor vessels; Chemotherapy delivery	Moderate (In vivo models)	Context-Dependent/Controversial	Opposes the traditional pro-angiogenic view; suggests potential anti-tumor role.	[129]
RNA/Protein Transfer (TEPs)	Biomarker signatures; Gene regulation in tumors	Moderate (Clinical sequencing data)	Emerging	Functional relevance of transferred RNA in vivo is still under investigation.	[123,130]
Apoptosis Induction (TRAIL)	Direct killing of tumor cells	Low/Moderate (Specific contexts)	Controversial	Contradicts the dominant pro-survival role of platelets; mechanism unclear.	[86,131]

## 6. Clinical and therapeutic implications

Understanding the multifaceted role of platelets in cancer progression has important clinical and therapeutic implications. Beyond their traditional function in hemostasis, platelets actively contribute to tumor growth, immune evasion, angiogenesis, and metastasis, making them attractive targets for both diagnostic and therapeutic strategies. Platelet-associated biomarkers may aid in early cancer detection, disease monitoring, and prognosis assessment, although their lack of absolute specificity remains a limitation. From a therapeutic perspective, antiplatelet agents and strategies targeting platelet–tumor interactions are being investigated as potential adjuvant approaches to limit cancer progression and metastasis. However, careful balancing of antitumor benefits with bleeding risk is essential in clinical translation.

### 6.1. Platelets as clinical biomarkers in cancer

Platelets have emerged as valuable indicators of cancer development, progression, and prognosis. Thrombocytosis, characterized by an increased platelet count, is commonly seen in various malignancies and is associated with poor clinical outcomes.^132^ The ability of platelets to reflect tumor activity is largely due to their interaction with cancer cells, stromal elements, and immune mediators, leading to their phenotypic and transcriptomic reprogramming.^81^ Consequently, tumor-educated platelets (TEPs) have garnered interest as non-invasive, blood-based biomarkers. Recent advancements in RNA sequencing have revealed TEPs as potentially promising non-invasive biomarkers for cancer detection. In a key study by Best et al., TEP mRNA profiles distinguished cancer patients from healthy individuals with high reported accuracy. Analyzed across 283 samples, 228 patients with localized or metastatic cancers, and 55 healthy donors, the TEP-based classifier achieved a pan-cancer detection accuracy of 96%. Although the accuracy for identifying specific primary tumor sites was 71%, these results highlight the dynamic RNA repertoire in platelets influenced by tumor presence. However, the biological mechanisms underlying platelet education and RNA transfer remain incompletely understood, and methodological variability between studies may affect reproducibility and clinical translation. To confirm these findings' clinical utility, further validation in larger, multicenter cohorts is essential to eliminate confounding factors, such as inflammatory comorbidities, and enhance cancer diagnostics.^123^ Elevated platelet counts have been linked to advanced tumor stages and poor survival outcomes in a range of solid tumors, including lung, breast, colorectal, pancreatic, and ovarian cancers.^133^ In non-small cell lung cancer (NSCLC), thrombocytosis has been associated with poorer survival outcomes. A meta-analysis of 37 studies involving 14,833 patients found that elevated platelet counts correlated with decreased overall survival (HR 1.033), disease-free survival (HR 1.568), and progression-free survival (HR 1.653).^134^ Similarly, in ovarian cancer, pre-treatment thrombocytosis (platelet count ≥ 400 × 10⁹/L) has been linked to worse prognosis. A pooled analysis of 14 studies with 5,414 patients revealed that thrombocytosis was associated with increased risk of recurrence (HR 2.01) and higher mortality (HR 2.29).^135^ Giannakeas et al. (2022) performed a nested case-control study on 8.9 million Ontario residents with routine CBC tests from 2007 to 2017, followed through 2018. Among them, nearly 495,000 were diagnosed with a first primary cancer. Platelet count percentiles were grouped by participants, and a very high platelet count (≥90th percentile) was associated with an increased risk of several solid tumors within 6 months of testing. The strongest associations were for colorectal (OR = 4.38), lung (OR = 4.37), ovarian (OR = 4.62), and gastric (OR = 4.27) cancers. These associations diminished as the time from test to diagnosis increased, indicating thrombocytosis may serve as an early hematologic marker for cancer risk stratification and early detection.^136^

Another example: Mounce et al. (2020) conducted a prospective cohort study using primary care electronic health records to evaluate cancer occurrence in patients with a normal platelet count, focusing on the high-normal range (326–400 × 10⁹/L).^137^ The study included 295,312 patients aged 40 years and older without prior cancer diagnoses, with oversampling of individuals with platelet counts above 325 × 10⁹/L to improve precision. Results showed that one-year cancer incidence increased with age, male sex, and higher platelet counts within the normal range. Notably, men aged 60 and older with high-normal platelet counts had significantly elevated cancer risk, reaching up to 6.7% in those over 80 with platelet counts between 376–400 × 10⁹/L. Lung and colorectal cancers were most commonly diagnosed, often at advanced stages. These findings suggest that high-normal platelet counts, particularly in older males, may serve as an early indicator of malignancy and warrant further clinical investigation; however, platelet counts may also be influenced by non-malignant inflammatory and age-related conditions, which may limit specificity.^138^ Also, Sylman et al. (2017) demonstrated that longitudinal analysis of platelet counts before and after cancer diagnosis provides valuable prognostic information, emphasizing the significance of thrombocytosis in cancer progression and patient outcomes.^132^ In contrast to prior studies linking thrombocytosis with advanced cancer stages and poor prognosis, Fahdrin et al. (2022) conducted a cross-sectional study of 171 breast cancer patients. They found no statistically significant association between platelet count and cancer stage as defined by the TNM classification (*p* = 0.952). Their results suggest that elevated platelet counts may not universally correlate with tumor burden or progression in breast cancer, highlighting the need for further investigation into cancer type-specific roles of platelet dynamics.^139^

The platelet-to-lymphocyte ratio (PLR) is another hematological index associated with systemic inflammation and tumor aggressiveness. Numerous studies have found that a high PLR correlates with advanced stage and decreased survival across cancer types. A large meta-analysis demonstrated that elevated PLR significantly predicted worse OS and DFS, suggesting its potential use in patient risk stratification.^140^ Nevertheless, PLR is a nonspecific inflammatory marker and may be influenced by a variety of non-cancer-related conditions. Beyond quantitative changes, platelets exhibit qualitative modifications in their RNA content. These RNA profiles are reported to be associated with specific cancer types and may enable non-invasive tumor detection. In a landmark study, TEP RNA signatures were able to distinguish six different cancers with over 95% sensitivity and specificity, highlighting their potential in early diagnosis and tumor classification. However, these findings still require validation across larger and more diverse patient populations, as well as standardized analytical approaches before routine clinical implementation can be considered.

Moreover, biomarkers of platelet activation, such as soluble *P*-selectin, PF4, and *β*-thromboglobulin, are frequently elevated in cancer patients. Although these biomarkers may reflect platelet activation associated with malignancy, they are not entirely cancer-specific and may also increase in inflammatory and thrombotic disorders. These factors not only promote tumor-associated thrombosis but also facilitate cancer cell extravasation and immune evasion. Thus, platelets are not mere passive observers but active participants in oncogenesis.^82^ ^,^ ^141^ ^,^ ^142^ In addition to quantitative alterations, recent findings highlight the role of platelet-derived molecules and receptors in mediating tumor–immune interactions. Notably, platelets can express immune checkpoint molecules such as PD-L1, which contribute to tumor immune evasion and may influence response to immunotherapy. PMPs, rich in VEGFR and *P*-selectin, have also been implicated in promoting angiogenesis and tumor progression. These molecular features further support the diagnostic and prognostic potential of platelet-based biomarkers beyond simple platelet counts.^61^ ^,^ ^120^ Nevertheless, the clinical applicability of these biomarkers remains under investigation, and additional mechanistic and prospective validation studies are required.

### 6.2. Therapeutic implications: balancing efficacy and safety

The therapeutic targeting of platelets in cancer represents a promising yet inherently complex strategy, with outcomes that are highly dependent on tumor type, disease stage, and patient-specific risk factors. While substantial preclinical and epidemiological evidence supports a role for antiplatelet agents in reducing metastasis, these benefits are not uniform across cancer types or patient populations. Importantly, the clinical applicability of such strategies is constrained by a well-established risk of bleeding, particularly gastrointestinal and intracranial hemorrhage, which must be carefully weighed against potential anti-tumor effects.

Current research distinguishes between two main clinical contexts: primary prevention, aimed at reducing cancer incidence in at-risk individuals, and adjuvant therapy, which seeks to limit metastatic progression in patients with established disease. In both settings, therapeutic efficacy appears to be highly context-dependent, with the strongest evidence observed in gastrointestinal malignancies, while results in non-GI cancers remain inconsistent.

#### 6.2.1. Primary prevention settings

Extensive epidemiological studies, particularly in colorectal cancer (CRC), suggest that long-term low-dose aspirin use may reduce cancer incidence and mortality by approximately 20–30%.^143^ ^,^ ^144^ However, this effect typically requires prolonged exposure (5–10 years) and is not consistent across all populations. The ASPREE trial demonstrated that in healthy elderly individuals (>70 years), aspirin did not confer a survival benefit and was instead associated with increased bleeding risk and higher overall mortality.^145^ These findings highlight that the risk-benefit ratio becomes unfavorable in older populations, emphasizing the need for careful patient selection.

The strongest preventive effects have been observed in high-risk hereditary populations, such as patients with Lynch syndrome. In the CAPP2 trial, high-dose aspirin significantly reduced colorectal cancer incidence in this group, indicating that genetic background is a critical determinant of therapeutic efficacy. Beyond colorectal cancer, some evidence suggests potential risk reduction in esophageal, gastric, and pancreatic cancers; however, these findings are less consistent and require further validation.^143^ ^,^ ^144^Overall, primary prevention strategies must account for chemopreventive lag time, bleeding risk, and inter-individual variability, particularly in elderly patients.

#### 6.2.2. Adjuvant therapy and metastasis prevention

In the adjuvant setting, the primary goal shifts toward preventing survival and dissemination of CTCs. Antiplatelet agents such as aspirin and P2Y12 inhibitors may disrupt platelet–CTC interactions, thereby reducing immune evasion and enhancing susceptibility to shear stress and NK-cell-mediated clearance. However, most supporting evidence derives from post hoc analyzes of cardiovascular cohorts, and prospective oncology trials remain limited.^76^ ^,^ ^146^ Meta-analytical data suggest that aspirin may reduce the risk of distant metastasis by 30–40% in adenocarcinomas, with effects emerging earlier in the disease course compared to primary prevention.^138^ Nevertheless, these benefits are not observed uniformly across tumor types. Recent evidence further supports a central role of platelet-derived thromboxane A2 (TXA2) in linking platelets to metastatic immune escape. Yang et al. ^147^, (Nature, 2025) demonstrated that TXA2 suppresses T cell activation via an ARHGEF1-dependent pathway, thereby promoting metastatic progression. Inhibition of COX-1 signaling or genetic deletion of ARHGEF1 restored T cell activity and significantly reduced metastatic burden in experimental models. These findings provide mechanistic support for aspirin’s anti-metastatic effects but also underscore that therapeutic efficacy depends on intact immune–platelet interactions and tumor-specific immune contexts.

An important new idea in adjuvant therapy is called precision prevention. Not every patient gets the same benefit. For example, in colorectal cancer, adjuvant aspirin is most effective in tumors with PIK3CA mutations. This mutation acts as a predictive biomarker because aspirin reduces PI3K signaling activity, thereby helping to stop tumor growth in this specific group.^139^ The perioperative period presents a critical window for therapeutic intervention. Although surgical resection is potentially curative, it induces a systemic inflammatory response and reactive thrombocytosis, which may inadvertently promote the survival of residual micrometastases. Evidence from preclinical models indicates that perioperative antiplatelet therapy can substantially reduce metastatic seeding.^82^ ^,^ ^148^ ^,^ ^149^ Ongoing clinical trials, including the Add-Aspirin study (NCT02804815), are expected to clarify the role of aspirin in adjuvant cancer therapy. Importantly, results to date suggest that any therapeutic benefit is likely to be tumor-type specific, with stronger effects in gastrointestinal cancers and less consistent outcomes in other malignancies.^150^

#### 6.2.3. Risk stratification: bleeding, stage, and site

The clinical translation of antiplatelet therapy in oncology requires careful balancing of anti-metastatic potential against bleeding risk. Long-term aspirin use is associated with a 40–50% increased risk of major gastrointestinal and intracranial hemorrhage, making safety a primary limiting factor. This risk is particularly pronounced in older individuals, in whom the absolute harm may outweigh potential oncologic benefit. Current guidelines, therefore, recommend that the optimal therapeutic window for initiating prophylaxis is likely between the ages of 50 and 59. Therapeutic efficacy is also strongly stage-dependent. Antiplatelet agents appear most effective in early and micrometastatic disease settings, particularly in the adjuvant context, whereas their benefit in advanced metastatic disease is limited. In addition, tumor-type specificity is a major determinant of response: the strongest evidence supports benefit in gastrointestinal cancers (colorectal, gastric, and esophageal), whereas evidence in non-GI tumors remains inconsistent.^144^ Given these limitations, future therapeutic strategies should prioritize precision medicine approaches incorporating tumor molecular profiling, anatomical site, stage of disease, and individualized bleeding risk assessment. Rather than a uniform application, antiplatelet therapy should be considered a context-dependent intervention with a narrow but potentially meaningful therapeutic window.

## 7. Conclusion

Platelets represent critical yet paradoxical players in oncogenesis and advancement. Their ability to promote tumor angiogenesis, immune evasion, and metastasis underscores their importance as therapeutic targets. However, the emerging evidence of platelet-mediated anti-tumor effects complicates the development of antiplatelet therapies and necessitates a nuanced understanding of platelet biology in different cancer contexts. Advances in molecular profiling of tumor-educated platelets and targeted inhibition of platelet-tumor interactions offer potential pathways for early cancer identification and precision treatment. Subsequent investigations must concentrate on delineating the organ- and stage-specific functions of platelets, optimizing the safety and efficacy of antiplatelet agents, and integrating platelet biomarkers into clinical practice. Harnessing platelets' dualistic nature could revolutionize cancer diagnostics and therapeutics, ultimately improving patient outcomes. Future clinical strategies must account for the heterogeneity of platelet function across different tumor types to ensure personalized, effective interventions.

## Data Availability

Data sharing is not applicable to this article as no data were created or analyzed in this study.

## Dedication

This manuscript is dedicated to my beloved 9-month-old son, Ayhan, whose presence, smiles, and gentle spirit brought light to my writing journey. I am sincerely grateful to him for giving me the precious time and motivation needed to complete this work.

## Abbreviations

ADP: Adenosine Diphosphate
ATP: Adenosine Triphosphate
bFGF: Basic Fibroblast Growth Factor
CAF(s): Cancer-Associated Fibroblast(s)
CD226: Cluster of Differentiation 226
CTC(s): Circulating Tumor Cell(s)
CTL(s): Cytotoxic T Lymphocyte(s)
DC(s): Dendritic Cell(s)
ECM: Extracellular Matrix
EMT: Epithelial-Mesenchymal Transition
GITRL: Glucocorticoid-Induced TNF-Related Ligand
GPCR(s): G-Protein-Coupled Receptor(s)
GPIb: Glycoprotein Ib
HMGB-1: High-Mobility Group Box-1
JNK: c-Jun N-terminal Kinase
MAPK/ERK: Mitogen-Activated Protein Kinase / Extracellular Signal-Regulated Kinase
MHC-I: Major Histocompatibility Complex Class I
MMP(s): Matrix Metalloproteinase(s)
NF-κB: Nuclear Factor Kappa-light-chain-enhancer of Activated B Cells
NKG2D: Natural Killer Group 2, Member D
NK cell(s): Natural Killer Cell(s)
OS/DFS: Overall Survival/Disease-Free Survival
PAR(s): Protease-Activated Receptor(s)
PDGF: Platelet-Derived Growth Factor
PF4: Platelet Factor 4
PI3K: Phosphoinositide 3-Kinase
PKC: Protein Kinase C
PlGF: Placental Growth Factor
PMV(s): Platelet-derived Microvesicle(s)
PMP(s): Platelet-derived Microparticle(s)
PSGL-1: P-Selectin Glycoprotein Ligand-1
RANKL: Receptor Activator of Nuclear Factor Kappa-B Ligand
ROS: Reactive Oxygen Species
SDF-1: Stromal Cell-Derived Factor 1
Smad: SMA and MAD-related protein family in TGF-β signaling
TGF-β: Transforming Growth Factor-beta
TEP(s): Tumor-Educated Platelet(s)
TKI(s): Tyrosine Kinase Inhibitor(s)
TLR4: Toll-Like Receptor 4
VEGF: Vascular Endothelial Growth Factor
VEGFR: Vascular Endothelial Growth Factor Receptor
vWF: von Willebrand factor
